# Time-Driven Activity-Based Costing (TDABC) applied in a chemotherapy department of a public reference oncology hospital

**DOI:** 10.31744/einstein_journal/2025GS0679

**Published:** 2025-07-23

**Authors:** Amanda Oliveira Serra-Campos, Camila Campos Valério, Liliane Rosa Alves Manaças, Marina Magnago Cruz, Rodrigo Saar da Costa

**Affiliations:** 1 Instituto Nacional de Câncer - INCA Rio de Janeiro RJ Brazil Instituto Nacional de Câncer - INCA, Rio de Janeiro, RJ, Brazil.

**Keywords:** Micro-costing, Costs and cost analysis, Antineoplastic agents, Drug therapy, Medical oncology, Health care costs, Technology assessment, biomedical, Health services, Unified health system

## Abstract

**Objective:**

To identify the costs pertaining to the preparation and administration process of each chemotherapy treatment session in a public oncology hospital belonging to the Brazilian Unified Health System.

**Methods:**

This prospective, observational, and descriptive study employs the bottom-up micro-costing method through Time-Driven Activity-Based Costing, from the perspective of the facilities of a chemotherapy department within a public oncology hospital.

**Results:**

A comprehensive overview of the flow related to outpatient antineoplastic therapy was elaborated through process mapping, which included each step required for a treatment session. Total personnel costs were calculated at R$ 287,66, distributed among the nursing (48.81%), pharmacy (18.66%), clinical analysis (16.27%), and clinical oncology (16.27%) sectors. The total cost of one intravenous antineoplastic therapy session per patient, excluding the cost of the antineoplastic drug, was R$ 470,35. Nursing care accounted for 49.88% (R$ 234,61) of the financial resources used per session, while pharmacy, clinical analysis, and clinical oncology accounted for 24.47%, 15.70%, and 9.95% of the costs, respectively.

**Conclusion:**

Employing the Time-Driven Activity-Based Costing method in health services not only facilitates resource optimization, providing quality and efficient care within constrained budgets, but also enhances control over processes and their financing.

## In Brief

The increasing incorporation of technologies in the health sphere and the limited availability of resources for service provision necessitating studies to estimate healthcare costs. Chemotherapy is one of the main cancer treatment options representing a high cost to the health system, making it essential to understand these costs for the efficient and equitable allocation of available resources.

## Highlights

■ Using the Time-Driven Activity-Based Costing method in healthcare services enables resource maximization with better control of processes and financing.
■ The estimated cost of treating a patient per antineoplastic session is R$ 470,35.
■ Such analyses can help establish pricing strategies and identify necessary adjustments to the financing model in oncology.

## Graphical abstract

**Figure:**
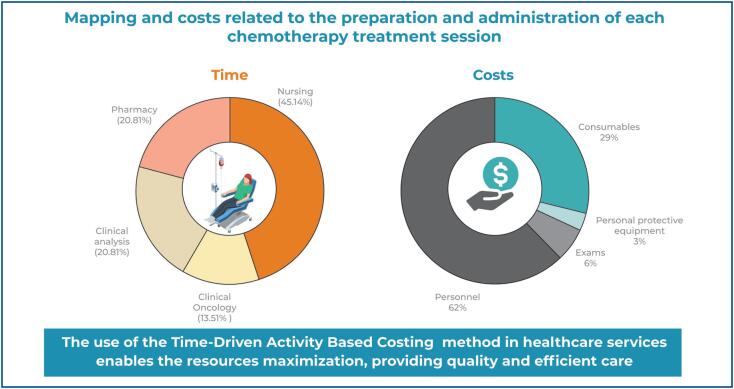

## INTRODUCTION

The increasing incorporation of high-cost technologies in the health sphere and the limited availability of resources for service provision highlight the need to conduct studies to estimate healthcare costs.^(1,2)^ Identifying, quantifying, and valuing all resources used during a given period in a therapeutic alternative program or health service is the objective of health cost analysis. Thus, the cost of an activity represents the monetary value of all organizational resources consumed to perform it.^(3)^ The evaluation of health costs involves using methods with two main characteristics: a way of identifying costs and a way of evaluating them. While costs can be identified through macro- or micro-costing methods, they can be estimated using the bottom-up or top-down approach.^(4)^

The bottom-up micro-costing method is considered the gold standard for economic evaluations in health because of its high degree of detail and accuracy in estimating direct and indirect costs, based on individual data from all resources used in the provision of an intervention. However, its implementation can be challenging owing to the lack of standardization of cost data collection methods and institutional particularities that hinder the comparison of the results obtained.^(5)^

Since the 1980s, the health sphere has used some micro-costing methods, such as absorption costing and Activity-Based Costing (ABC). However, the traditional ABC model can be challenging for many organizations to implement owing to the high costs of interviewing individuals for the initial survey of the method.^(6)^ In order to overcome these limitations, Kaplan and Anderson developed the Time-Driven Activity-Based Costing (TDABC) method in 2007.^(7)^

The TDABC method aims to measure the costs of health services according to the actual consumption of resources, thereby providing a more accurate estimate.^(4)^Through this method, the cost drivers can be converted into time equations that reflect the duration of each activity.^(8)^ The literature reveals that application of the TDABC method has become widespread. Despite being a complex method, TDABC is considered efficient, as time is a fundamental factor in estimating the costs of activities. Currently, the importance of the cost per minute of each resource used in the execution of an activity is widely recognized in the analysis of health costs. However, the implementation of TDABC can be challenging for many organizations because of the complexity of data collection.^(6)^

Economic analysis in oncology is crucial, as cancer is one of the greatest public health challenges worldwide and is among the top four causes of premature death in most countries. According to the National Cancer Institute (INCA - *Instituto Nacional de Câncer*), 704,000 new cases of cancer are expected to be reported each year from 2023 to 2025.^(9)^

Chemotherapy is one of the main cancer treatment options. However, these treatments may represent a high cost for the Brazilian Public Health System (SUS - *Sistema Único de Saúde*), making it essential to understand these costs for the efficient and equitable allocation of available resources.^(10,11)^ Studies of this nature are scarce in Brazil, especially in the field of oncology.

## OBJECTIVE

This study aims to identify the costs related to the preparation and administration process of each chemotherapy treatment session in a public oncology hospital belonging to the Brazilian Unified Health System, using the Time-Driven Activity-Based Costing method for a micro-costing analysis, measuring the costs involved in a chemotherapy session with a high level of detail.

## METHODS

This prospective, observational, and descriptive study employed the bottom-up micro-costing method through TDABC from the perspective of the facilities of a chemotherapy department of a public oncology hospital.

A mapping of the process related to outpatient chemotherapy in a period of less than 24 hours was structured, sequentially depicting all the steps taken by the patient, right from the medical appointment to the infusion of the antineoplastic drug. The activities/sectors involved in an antineoplastic chemotherapy cycle were identified: clinical oncology, clinical analysis, pharmacy, and nursing. This map was created to facilitate the understanding of the costs involved in the activities (phases of the clinical course), evidencing the value stream of the cancer patient.

The resources used in each activity during a session of intravenous antineoplastic chemotherapy were divided into personnel (doctors, nurses, pharmacists, and nursing or pharmacy technicians) and consumables (personal protective equipment [PPE], supportive drugs, supplies, and laboratory tests). The costs of antineoplastic drugs were not included in the analysis because of the large price discrepancy between them. Once the costs of the process are identified, the cost of the corresponding antineoplastic agent can easily be added. To determine the materials and medications needed for an intravenous antineoplastic treatment session, two protocols for metastatic melanoma were used as models: (1) chemotherapy with dacarbazine (long infusion) and (2) immunotherapy with pembrolizumab (short infusion). The cost per treatment session was based on the average of the supplies and medications used in both protocols, excluding antineoplastic drugs.

In addition, information about the workload of each professional involved in the activities was obtained. For each clinical visit, the following items were included in the costing exercise: medications (quantity used and unit price); supplies/examinations, PPE (quantity used and average unit price), and personnel (category, time in activity).

Salary costs were specified by distinguishing the activities performed by each professional category through the entire process, such as consultancy examinations, preparation, and administration of medications. The reference value for all professionals was obtained from the salary table of science and technology careers (SINDCT - *Sindicato Nacional dos Servidores Públicos Federais na Área de Ciência e Tecnologia do Setor Aeroespacial*), reference year 2022. The value calculated for the labor of a technologist (physician, nurse, or pharmacist) was R$ 1,56 per minute, while that for nursing and pharmacy technicians was R$ 0,76 per minute. The total personnel cost was calculated by multiplying the hourly wage of each employee by the total time invested in the activity.

For consumable costs, the corresponding quantities were recorded during process mapping and actual measurements. Items have been used at 100% capacity. The prices of PPE, medicines, and supplies were obtained from the hospital's internal system, based on the average value of the last three acquisitions occurring between the years 2017 and 2022. Total costs were calculated as the sum of the quantity multiplied by the prices of consumables.

To calculate the cost of PPE, the number of consumables used in a production cycle involving 25 antineoplastic bags being handled by the pharmacist and 12 patients being cared for by the nursing team was considered. The value of the basic examination package was derived from the SUS Procedures, Medications, and OPM Table Management System (SIGTAP - *Sistema de Gerenciamento da Tabela de Procedimento*), reference year 2022.

The source of the information defined for the valuation of each item was submitted to an evaluation of its degree of uncertainty, which varied between high, medium, and low. Data obtained from the institutional price database were classified as having a low degree of uncertainty, while data from the SIGTAP table were classified as having a high degree of uncertainty. A medium degree of uncertainty was assigned to items that aggregate data from SIGTAP and the institutional database, in addition to those linked to SINDCT, based on the hypothesis of the average remuneration of the human resources employed.

Capacity cost rates were obtained by dividing the cost of each resource by its practical capacity. The practical capacity of the care workforce was calculated based on the professionals’ monthly workload, divided into four-hour shifts. The number of hospital vacancies available for the administration of antineoplastic chemotherapy per shift was found to be equivalent to 12 sessions.

The times required for each activity were estimated based on *on-site* observation studies and time collection. To do this, the production of each activity was observed, and the average time (in minutes) required was calculated over two weeks during the operating hours of the Chemotherapy Department. The costs of all individual items and salary data were then collected to generate the aggregate unit costs by sector. The total costs of treatment were obtained by adding up the direct medical costs. The monetary data were processed and analyzed using Microsoft Excel^®^, version 2019.

To estimate the final cost of the clinical course of the cancer patient by a session of intravenous antineoplastic treatment, we considered the sum of the estimated costs of each resource (sum of the products of each resource by respective times of use).

Finally, to identify the effect of different hypothetical scenarios, the analysis was repeated with a univariate variation of the following parameters: the value of professional remuneration; inclusion of costs with antineoplastic drugs (dose calculated for patients 160cm in height and weighing 70kg); and maximum operating capacity of the Chemotherapy Department.

## RESULTS

The flow overview related to outpatient antineoplastic therapy was elaborated through process mapping and included each step required for a treatment session. On-site observations revealed the involvement of four main services in this process: clinical oncology, clinical analysis, pharmacy, and nursing. The process begins with a medical appointment in which the treatment protocol is defined according to the type of cancer, stage of the tumor, and clinical conditions of the patient. Next, laboratory or imaging tests are performed to monitor therapeutic response and toxicity related to antineoplastic therapy.

**Figure 1:**
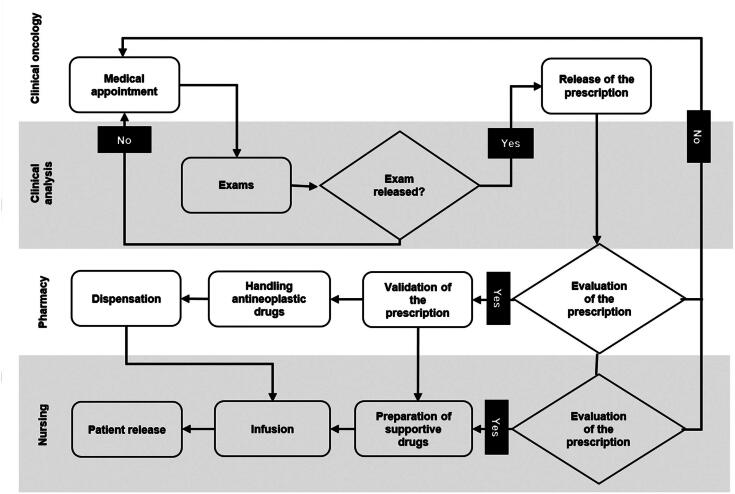
Mapping of the outpatient antineoplastic intravenous therapy process

The pharmacy team is responsible for evaluating the prescription (validation of data regarding the antineoplastic protocol, medication dosage, and patient), handling antineoplastic medications, and dispensing them to the nursing team. The nurses, in turn, evaluate the medical prescription and confirm the data pertaining to the medications received, prepare supportive medications (pre- and post-chemotherapy and hydration), and administer the antineoplastic agents to the patient. If any intercurrence is identified during the process, the patient is referred for medical reassessment (Figure 1).

The dilution center—a pharmacy sector—is responsible for producing approximately 25 chemotherapy bags per four-hour shift. The infusion room—a nursing sector—consists of 12 hospital seats that are occupied according to the time required for the infusion of each antineoplastic protocol, ranging from 30 (short infusion) to 180 (long infusion) minutes, which may include pre-chemotherapy, more than one bag of the antineoplastic agent, and venous hydration.

The evaluation of the activities involved in the antineoplastic therapy process allowed the creation of a detailed list of the resources used. Table 1 presents the identified types of cost and systematizes their valuation, respective data sources, and degree of uncertainty regarding the estimates.

**Table 1:** Valuation of procedures, services, and inputs consumed; data sources; and degrees of uncertainty regarding the estimates

Cost type	Definition	Valuation	Source	Degree of uncertainty
Medical appointment	Outpatient: cost of consultation/hour	Average time of the professional in the activity*	SINDCT	Medium
Laboratory tests	Package of examinations performed before each antineoplastic session. In addition, the cost of direct labor per exam was considered	Unified table of procedures, medicines, and strategic inputs of the SUS. Average time of the professional* &	SINDCT	Medium
Supportive drugs	Medications related to pre- and post-chemotherapy and hydration protocols	Average unit price/dose	Institutional price	Low
Input/consumable	Cost of all disposable materials used (IV catheter, needle, syringe, etc.) during the preparation and administration of the medications	Average unit price	Institutional price	Low
PPE/Clothing	PPE used during the preparation and administration of the antineoplastic agents, pre- and post-chemotherapy, and hydration	Average unit price	Institutional price	Low
Pharmaceutical service/nursing service preparation time/Administration (direct labor)	Salary and benefits of the clinical staff who validate prescriptions, provide patient care, and handle, administer, or dispense the antineoplastic agents and supportive drugs (*i.e.*, nurses, nursing assistants, pharmacists, and pharmacy technicians)	Average time of the activity professional/technologist*and technical level&	SINDCT	Medium

* Labor costs per minute of a technologist (doctor, nurse, or pharmacist): R$ 1,56;

& Labor costs per minute of a nursing or pharmacy technician: R$ 0,76.

The cost of labor was calculated from the salary perspective of science and technology careers (SINDCT).

PPE: personal protective equipment; SINDCT: *Sindicato Nacional dos Servidores Públicos Federais na Área de Ciência e Tecnologia do Setor Aeroespacial*.

At various stages of the process, healthcare professionals see more than one patient simultaneously. In these cases, the cost of the activity was calculated per work shift, with the cost of four hours of professional work being divided by the number of patients treated in that period.

In the evaluated institution, 2 doctors in each are responsible for the care of 16 patients, with an average medical appointment time of 30 minutes. In addition, in one shift, blood samples are collected and analyzed from 12 patients. The pharmacy analyzes approximately 12 medical records and produces 25 bags. Finally, 4 nurses are responsible for the care of 12 patients.

Total personnel costs were calculated at R$ 287,66, distributed among the nursing (48.81%), pharmacy (18.66%), clinical analysis (16.27%), and clinical oncology (16.27%) sectors. Personnel costs required the highest financial resources within the four sectors to treat one patient per antineoplastic therapy session.

**Figure 2:**
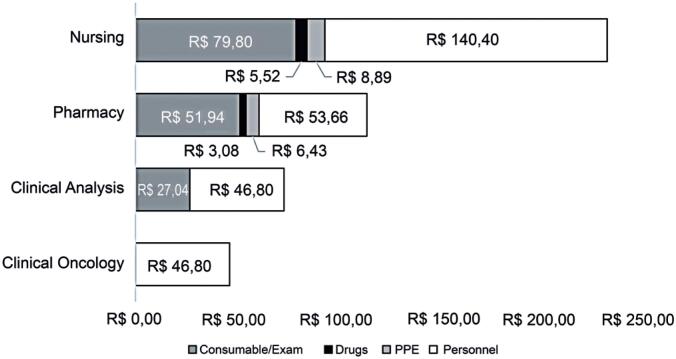
Distribution of personnel and consumable costs by sector for the treatment of an oncology patient

The total of consumable resources was R$ 182,69. Among these, the largest portion was allocated to inputs 28% (R$ 131,74), followed by the exam package at R$ 27,04 (5.75%), PPE at R$ 15,31 (3.26%), and medicines at R$ 8,60 (1.83%). Figure 2 represents the relative proportions for each type of cost associated with a session of antineoplastic therapy.

PPE: personal protective equipment.

**Figure 3:**
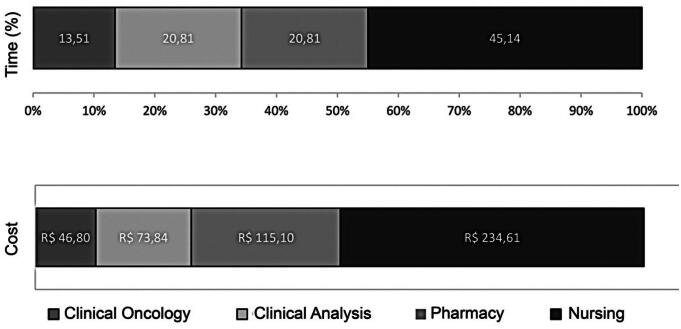
Composition of the total cost of the outpatient intravenous antineoplastic treatment in corresponded to an average time of 3.7 hours

The longest length of stay of the patient in the antineoplastic therapy process was in the nursing sector, averaging 1 hour and 40 minutes (45.14%), followed by the pharmacy (20.81%), clinical analysis (20.81%), and clinical oncology (13.51%) sectors. The estimated total time of the process was 3.7 hours (Figure 3A).

The total cost of one intravenous antineoplastic therapy session per patient, excluding the antineoplastic drug, was R$ 470,35 (Nursing care consumed 49.88% (R$ 234,61)) of the financial resources used per session, pharmacy, clinical analysis, and clinical oncology accounted for 24.47%, 15.70%, and 9.95% of the costs, respectively (Figure 3B).

Finally, the impact of a few cost parameters was evaluated, replacing the data according to different hypothetical scenarios. Considering that the science and technology career plan does not reflect the scenario of most professionals involved with these activities, the study reassessed this process using the Brazilian Guide of Occupations (GBO - *Guia Brasileiro de Ocupações*) (available at http://pdet.mte.gov.br/guia-brasileiro-de-ocupacoes, accessed February 20, 2023) as a reference. The value of time worked (in minutes) was recalculated for oncologists (R$ 1,35), nurses (R$ 0,49), hospital or clinical pharmacists (R$ 0,47), pharmacy technicians (R$ 0,24), and nursing technicians (R$ 0,23). The obtained total cost of personnel was R$ 115,01, 60% lower than the value of R$ 287,66 for science and technology careers.

**Figure 4:**
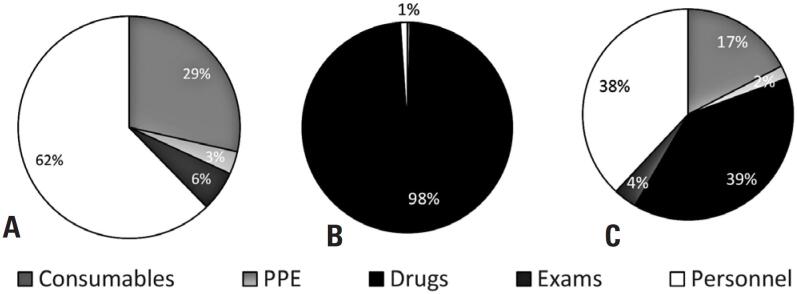
Total cost of the intravenous antineoplastic therapy. (A) without the cost of antineoplastic agents, (B) with short infusion (pembrolizumab), and (C) with slow infusion (dacarbazine)

To estimate the impact of antineoplastic drugs on process costs, two protocols for metastatic melanoma were compared: dacarbazine (systemic chemotherapy with a long infusion time) and pembrolizumab (high-cost immunotherapy with a short infusion time). Adding the antineoplastic cost to the analysis components reveals the pharmacy sector as the one with the greatest economic impact on the total costs of a cancer patient's treatment, especially with regard to high-cost drugs, such as pembrolizumab (Figure 4).

PPE: personal protective equipment.

When production capacity is doubled, reaching the maximum productivity limit of the Department of Chemotherapy per shift, without changing the number of professionals involved, the total cost of one session of chemotherapy per patient exhibits a decrease. The total cost went from R$ 470,35 to R$ 316,14, a reduction of 32.8%. The impacts were mainly on the cost of personnel and PPE, as more patients would be benefiting from these resources simultaneously.

## DISCUSSION

The present study estimated the average cost of the outpatient intravenous antineoplastic therapy process through bottom-up micro-costing using TDABC. The mapping of the process and the calculation of the time spent in each activity was conducted through on-site observations of each stage of intravenous antineoplastic therapy. Because it is a complex process that involves different sectors and professional categories, the interview method can introduce bias owing to segmented information and additional costs.

Variations in the application of the TDABC method among published studies pose challenges for reproducibility and data comparison. Nonetheless, most studies adhere to the seven-step model, where the starting point involves selecting the medical condition to be mapped or addressing the research question.^(12-15)^

Dossin et al. collected time data by taking three measurements on different days—with an additional three measurements taken in cases of significant time variability—using their median as the activity time.^(16)^ Lopes et al. gathered activity times through interviews with sector employees, later validating the data by monitoring patient care with timing.^(17)^ Nagra et al. estimated activity times using innovative information technology, validating them through patients’ treatment observations,^(18)^ using a Real-Time Location System that tracks the movements of patients and their team in clinical areas to capture "event" times, such as the patient's arrival at check-out.^(18-21)^ Thus, even in studies employing interviews for data collection, a stage of observation and monitoring of the process is used to validate the results.

Concerning the costs of resources for patient care, studies vary in terms of whether they include primary resources (direct care costs) and/or those needed for support (indirect costs).^(13)^ In this study, only direct costs were considered. Personnel costs are commonly calculated based on annual salaries or hours worked. Because indirect costs and support resources are viewed as fixed costs beyond the department's sphere of influence, analyses of these data with an internal focus may not be beneficial.^(13)^

In this study, the cost of an outpatient intravenous antineoplastic therapy session was estimated to be R$ 470,35. In 2019, Sohi et al., after reviewing 44 studies on chemotherapy administration, arrived at an average cost for the administration of intravenous chemotherapy of US$ 142/hour.^(22)^ These values do not include the antineoplastic agents and the management of adverse effects. However, in our study, the total cost was not calculated per hour but rather for the antineoplastic session process, which includes consultation, tests, and the preparation and administration of the antineoplastic agent. Sohi's review included studies with different methodologies for cost analysis, making it difficult to compare the results.^(22)^

Capacity estimates for each resource are commonly obtained through interviews. Some articles adjust theoretical capacity fixed rates for intervals, education, teaching, and research. Ignoring these adjustments may lead to the underestimation of the capacity cost rate (CCR) and, therefore, care processes costs.^(13)^ Zanotto et al., revealed a significant divergence in the median time and CCR for each resource in each phase of care for the same procedure across different hospitals in Brazil.^(23)^ In our study, considering that a science and technology career plan may overestimate personnel costs, a cost adjusted to the average market scenario was adopted. This salary change generated a 60% reduction in personnel costs (from R$ 287,66 to R$ 115,01), and a 36.71% decrease in the total process cost (from R$ 470,35 to R$ 297,70). This may be explained by the variability in contracts among regional macroeconomic conditions.^(13)^

According to the cost categories, personnel expenses (human resources) accounted for the largest share of total process costs. This corroborated the data by Lopes et al., who measured the costs for main immunobiological infusion procedures in the Center for the Dispensation of Medications by applying the TDABC method.^(17)^ Both studies considered an eight-hour work capacity per day. However, in specific treatments requiring high-cost drugs (as in medical oncology) or devices (as in mastectomies with implant reconstruction), these costs constitute the major share, surpassing personnel costs.^(18)^ This was observed in our study through an analysis incorporating the high-cost antineoplastic agent pembrolizumab—a monoclonal antibody used for the treatment of metastatic melanoma.

The institution studied is a referral unit for gynecological cancer and bone/connective tissue tumor treatment. In 2021, 1,089 active patient enrollments were recorded and 5,871 chemotherapy sessions were performed.^(24)^ Therefore, according to our data, it is possible to estimate an annual cost of 2.761.424,85 (R$ 470,35/session) for outpatient intravenous antineoplastic therapy.

The advent of immunotherapy and molecular-targeted therapy brought positive gains for the survival of cancer patients but significantly increased oncology costs. Thus, although costs with antineoplastic agents have been widely evaluated in cost-effectiveness studies, research focusing on the analysis of antineoplastic therapy processes are scarce. Our results underscore that the process costs should not be overlooked, as they constitute a significant portion of overall expenses. In this work, we isolated the process costs and defined measurable elements, enabling the model's application in various scenarios, evaluating the impact of each element—including antineoplastic agents, which canbe easily incorporated into the process calculation.

The main limitation of this study is the use of average cost for elements that have variable characteristics, such as inputs and the salaries of healthcare professionals. However, one of its strengths is the identification of parameters predictive of high patient treatment costs, which is useful for decision-making. In addition, the non-incorporation of the values related to the structure in costing evaluations allows for the application of the analysis to other service units.

Several economic analyses in healthcare aim to estimate the cost of clinical procedures, enable comparisons with health plan reimbursement rates, and identify points of operational improvements.^(13,16,17)^ Nevertheless, it is important to emphasize that the patient's characteristics influence process costs. Nagra et al. evaluated comorbidities and interventions for breast cancer patients, revealing that personnel expenses primarily contributed to the cost of surgical care, except for mastectomies with implant reconstruction, where the cost of the device accounted for up to 60% of the cost of the surgery.^(18)^ These findings align with our analysis, which revealed the impact of specialized human resources on the cost of oncological activities. In addition, they reinforce how including a single technology in the process—such as implants—can affect service costs.

The financing model for procedures related to antineoplastic treatment is based on reimbursement through a fixed table established for each protocol and tumor type. This approach for highly complex care is known as the *Autorização de Procedimento Ambulatorial* (APAC). However, its value is often lower than the actual treatment costs. Furthermore, APAC rates are considered a limiting factor for the implementation of new technologies.^(25)^ Findings from this studies can help establish pricing strategies and identify necessary adjustments to the financing model. However, future studies focusing on economic analysis models for oral treatments are essential.

In 2023, Law No. 14,758 was enacted, establishing the National Policy for Cancer Prevention and Control within the scope of the SUS. This law introduced significant changes to the APAC financing model.^(26)^Currently, an example of new perspectives in the APAC model can be observed in the financing of treatments for children and adolescents with acute lymphoid leukemia. This includes reimbursement for the exchange of continuous infusion bags at a value of R$496.90, covering the chemotherapy manipulation process. Additionally, an exclusive APAC for new drugs used in the treatment is available, compatible with market values.^(25,27)^ It is important to highlight that the value established for this APAC was similar to that observed in our study, excluding medication costs.

Finally, these results can contribute to future economic decision analyses in healthcare and support initiatives promoted by the Ministry of Health. These initiatives aim to adapt the fee-for-service model via APAC to accommodate the introduction of high-cost technologies for cancer treatment while also consolidating long-term savings.

## CONCLUSION

Using the Time-Driven Activity-Based Costing method, we mapped the process and estimated the cost of treating individual patients per antineoplastic session at R$ 470,35. Personnel costs constituted the greatest impact on care costs, except in treatments that required high-cost medications, such as pembrolizumab, as revealed by the analysis of hypothetical scenarios. Administration and preparation processes need to be carefully evaluated alongside chemotherapy costs in health technology assessment and reimbursement agreements. The use of the Time-Driven Activity-Based Costing method in healthcare services enables resource maximization, providing quality and efficient care, with better control of processes and financing, in the face of limited budgets.
